# Impact of water fortification with calcium on calcium intake in different countries: a simulation study

**DOI:** 10.1017/S1368980020002232

**Published:** 2022-02

**Authors:** Gabriela Cormick, Luz Gibbons, Jose M Belizán

**Affiliations:** 1 Department of Mother and Child Health Research, Institute for Clinical Effectiveness and Health Policy (IECS-CONICET), Buenos Aires 1414, Argentina; 2 Departamento de Salud, Universidad Nacional de La Matanza, San Justo 1754, Argentina

**Keywords:** Calcium, Fortification, Intake, Simulation, Water

## Abstract

**Objective:**

To simulate the impact – effectiveness and safety – of water fortification with different concentrations of Ca using the Intake Modelling, Assessment and Planning Program.

**Design:**

This is a secondary analysis of national or sub-national dietary intake databases.

**Setting and Participants:**

Uganda, Lao People’s Democratic Republic (PDR), Bangladesh, Zambia, Argentina, USA and Italy.

**Results:**

We found that for dietary databases assessed from low- and middle-income countries (LMIC), the strategy of fortifying water with 500 mg of Ca/l would decrease the prevalence of low Ca intake in all age groups. We also found that this strategy would be safe as no group would present a percentage of individuals exceeding the upper limit in >2 %, except women aged 19–31 years in Lao PDR, where 6·6 % of women in this group would exceed the upper limit of Ca intake. The same strategy would lead to some groups exceeding the upper limit in USA and Italy.

**Conclusions:**

We found that for most LMIC countries, water fortified with Ca could decrease the prevalence of Ca intake inadequacy without exceeding the upper levels of Ca intake.

Adequate Ca intake is associated with bone health, and increasing evidence shows the link between adequate Ca intake and lower blood pressure particularly among young people, the prevention of hypertensive disorders of pregnancy and lower blood pressure in the progeny of mothers taking sufficient Ca during pregnancy^(1–5)^. Mechanisms linking low Ca intake and blood pressure are mediated by parathyroid hormone raise that increases intracellular Ca in vascular smooth muscle cells leading to vasoconstriction^(6)^. An adequate Ca intake has also shown to lower cholesterol values and to prevent renal stones and colorectal adenomas^(7–10)^.

Ca intake is low in many countries in the world, in particular, in low-income countries, although low Ca intake can also be found within some population age groups in certain high-income countries (HIC)^(5,11–15)^. The promotion of diets with Ca-rich foods is the ultimate goal. However, a changing dietary habit is difficult to achieve in the short term. In countries where inadequate Ca intake is widespread, Ca supplements might not be the best option, as they require good adherence, which is often difficult to be achieved by the whole population^(16)^. Furthermore, it has been shown that supplementation adherence is usually better in individuals who already have better micronutrient intake, leading to an improvement of the population diet but not necessarily in those more at need^(17)^. Therefore, food fortification strategies seem to be a good option, although they require appropriate planning to determine the food fortification level necessary to improve Ca intake without putting any population group at risk of excess^(18)^. Fortification planning requires data on food and nutrient intake representative of the aimed population so as to define the level of nutrient inadequacy in the population and then decide the appropriate food vehicle that can be fortified^(18)^. The next step is a simulation of the impact of different fortification levels on the distribution of nutrient intake of each population group that will be reached by the fortification strategy. This step allows to estimate the reduction of nutrient inadequacy for each population group as well as the percentage of individuals with excess intake.

Traditionally, fortification vehicles have been staple foods and condiments such as salt as these foods are vastly consumed ensuring to reach all individuals in a given population. Although less explored as a fortification vehicle, water has been used in fluoride fortification^(19)^. Ca fortification of water seems a promising approach as water is universally consumed and Ca in water has a good bioavailability^(20–22)^. Ca bioavailability from Ca-rich waters is similar to that of milk^(20–22)^. Ca absorption might be increased in certain groups such as pregnant women, for that reason some countries do not increase the recommendation of Ca intake^(23)^. Furthermore, Ca bioavailability is improved when Ca intake is spread in low doses throughout the day rather than in one load. In this way, water as a vehicle seems a good alternative even if the Ca content in water is not very high^(24)^. Another benefit of using water as a fortification vehicle is that increasing water intake is also a beneficial public health promotion message, whereas other vehicles such as salt or wheat flour might have some consumption restrictions for certain population groups with hypertension or food allergies. Even more, increasing water intake is part of a healthy diet and taking into account the global obesity epidemic increasing water intake does not comprise a change in the total energy intake.

The IOWA University has developed the Intake Modelling Assessment Program (IMAPP), a computer programme that allows running the simulation of different fortification scenarios using the information of population daily nutrient intake. The programme allows estimating the best amount of a fortificant to be added to a food vehicle in order to decrease the level of nutrient inadequacy, without exceeding the recommended upper limits^(25,26)^.

The objective of the current study is to simulate the impact – effectiveness and safety – of water fortification with Ca using the available dietary intake information from Uganda, Lao People’s Democratic Republic (PDR), Bangladesh, Zambia, Argentina, Italy and USA^(11)^. These countries show a diversity of situations involving low- and middle-income countries (LMIC), one from South America, two from Africa, two from Asia and two HIC, one from North America and one from Europe.

## Materials and methods

We search for available national or sub-national dietary assessment databases collected through 24-h recalls or dietary records, we analysed all publicly available. This is an analysis of dietary intake databases of 24-h recalls from Uganda, Lao PDR, Bangladesh, Zambia, Argentina and USA and of self-recorded food records from Italy. Uganda, Lao PDR, Bangladesh, Zambia and Argentina are countries in regions with very low Ca intake (below 400 mg/d), whereas the USA and Italy show an overall adequate Ca intake^(11)^. Databases from Uganda, Lao PDR, Bangladesh, Zambia and Italy were obtained from the FAO/WHO Global Individual Food consumption data Tool, an open-access online platform, hosted by FAO and supported by WHO^(27)^. Argentina’s database was obtained from the Ministry of Health, and the USA’s database was obtained from the Centers for Diseases Control and Prevention website^(28,29)^.

For each database, we evaluated the effectiveness and safety of different Ca fortification scenarios with the aim of shifting the distribution of Ca intake so that the majority of population groups improve their Ca intake and achieve a mean intake closer to their requirement without exceeding the recommended upper limit^(25)^. Effectiveness was measured as the percentage of individuals below the Institute of Medicine (IOM) estimated average requirement (EAR) and safety as the percentage of individuals exceeding the IOM upper limit (UL) of their corresponding age-specific population subgroup.

The Ca EAR defined by the IOM is 270 mg/d for infants <1 year, 400 mg/d for children aged 1 to <4 years, 640 mg/d for children aged 4 to <9 years and 1100 mg/d for those aged 9 to <19 years. The EAR for Ca defined by the IOM is 800 mg/d for those aged 19 to <51 years, 800 mg/d for males aged 51 to <71 years and 1000 mg/d for women aged ≥51 years and men ≥71 years. The Ca UL defined by the IOM is 1500 mg/d for infants <1 year, 2500 mg/d for children aged 1 to <9 years, 3000 mg/d for those aged 9 to <19 years, 2500 mg/d for those aged 19 to <51 years and 2000 mg/d for those aged ≥51 years. Pregnant and lactating women aged 14–50 years have the same EAR and UL as those of non-pregnant women.

### Population

#### Uganda

We used dietary intake data from the baseline survey of the HarvestPlus Reaching End Users Orange-Fleshed Sweet Potato project performed in three rural regions of Eastern and Central Uganda: Bukedea, Kamuli and Mukono. This cross-sectional study too place from 1st January to 31st December 2007. The project aimed at inducing broad Orange-Fleshed Sweet Potato adoption to increase vitamin A intakes and reduce vitamin A deficiency among women in Uganda^(27)^. The survey included 577 women of reproductive age. After classifying participants into the IOM age categories for dietary recommended values, we found that only three girls were younger than 19 years, so they were excluded from the analysis. Nine of those had missing data, and they were also excluded. A total of 565 women were included in the dietary intake analysis, 270 non-pregnant women aged 20–67 years and 295 pregnant women aged 19–48 years.

#### Lao People’s Democratic Republic

We used data from the National Food Consumption Survey Lao People’s Democratic Republic performed in rural and urban settings from December 2016 to May 2017 to cover both the dry and rainy seasons^(27)^. The survey included 2045 individuals, 870 males and 1175 women. According to the IOM age categories, we found a few number of individuals in the groups of pregnant women aged 51 or above, non-pregnant women aged 71 or above and boys aged 9 to <19 years so they were excluded from this analysis. A total of 1919 individuals were analysed in the current study, 1048 children aged 3 months to <14 years, 319 non-pregnant girls and women aged 14–86 years, 285 pregnant women aged 16–56 years and 267 men aged 19–89 years.

#### Bangladesh

We used data from the initial survey of a HarvestPlus multi-stage research programme to determine the potential impact of Zn-biofortified rice on the Zn and health status among children in Bangladesh. The original study was conducted in collaboration with the University of California, Davis and the International Centre for Diarrhoel Disease Research, Bangladesh (ICDDR) from 30th September 2007 to 29th June 2008. Dietary information was collected primarily by direct observation and weighing of food preparation and consumption^(27)^.

According to the IOM age categories, there were 224 non-pregnant women aged 18–70 years and 236 pregnant women aged 5–50 years. The survey also included girls aged 14–19 years and women aged 51 to <71 years; however, as there were very few cases in those groups, we were not able to analyse them.

#### Zambia

We used data from the initial survey of the HarvestPlus nutritional survey. This survey was carried out in Nyimba District in Eastern Province and Mkushi District in Central Province with the collaboration with the National Food and Nutrition Commission (Lusaka, Zambia) and the Tropical Diseases Research Centre from 30 April 2007 to 29 December 2009. The main goal of the survey was to obtain adequate background nutritional information among preschool children from rural Zambia to assess the potential impact of food-based interventions to improve vitamin A status, including provitamin A-biofortified maize^(27)^.

According to the IOM age categories, there were 454 children aged 1 to <9 years, 145 non-pregnant women aged 19 to <50 years and 186 pregnant women aged 17 to <51 years. The survey also included infants <1 year and girls aged 14 to <19 years (pregnant or not) and women (pregnant or not) aged 51 to <71 years; however, there were very few cases in those groups and we were not able to analyse them.

#### Argentina

We used data from the first Health and Nutrition National Survey carried out by the Ministry of Health in Argentina between 2004 and 2005^(28)^. Participants were selected using a probabilistic complex sample design including different socioeconomic levels from both large and small cities of all provinces of Argentina representing the whole population^(30)^. Data were collected using a single 24 h recall.

The survey included 1338 children aged 0 to <9 years, 6605 women aged 19 to <50 years and 1610 pregnant women 14 to <45 years.

#### Italy

We used data from the third national survey L’indagine nazionale sui consumi alimentari in Italia performed by the Italian Consiglio per la ricerca in agricoltura e l’analisi dell’economia agrarian from October 2005 to December 2006^(31)^. The survey covered all seasons and included a sample representative of the North-West, North East, Centre, South and Islands of Italy. Consumption of all foods, beverages, food supplements and medicines was self-recorded by subjects for three consecutive days on hard-copy diaries structured by meal. For our analysis, we used the first and third survey days.

The survey included 3323 individuals 1501 men and 1793 women; however, some had not all required data, and finally, 3269 were analysed. According to the IOM age categories, there were 377 children aged 0 to <14 years, 28 pregnant women aged 19 to <51 years, 1584 non-pregnant women aged 19 to <97 years and 1280 men aged 19 to <92 years.

#### USA

We used data from the 2016 nationally representative, cross-sectional survey of the non-institutionalised US population National Health and Nutrition Examination Survey (NHANES) administered by the National Center for Health Statistics within the CDC^(29)^. The survey has a stratified, multistage probability cluster sampling design.

The survey included 9971 individuals, however, 8339 had data on Ca intake, 2443 children aged 0 to <19 years, 2974 women aged 19 to <80 years, 2859 men aged 19 to <80 years and sixty-three pregnant women 20 to <42 years.

### Analysis

Ca intake was estimated using the daily intake information from individuals in each of the selected countries. Each individual was classified according to the IOM dietary reference values age-specific groups: children, adolescents, adults and pregnant women. Afterwards, we determined how many individuals were in each population group and how many had repeated dietary intake information.

We estimated day-to-day Ca intake variability of for those groups that had a repeated dietary intake data in non-consecutive days. The variability was then used to adjust the Ca intake distribution of one single day in order to obtain an estimated distribution of Ca usual intake. If repeated dietary intake data were not available, we estimated the usual Ca intake using a default external variance ratio for Ca provided by the IMAPP.

We used IMAPP to calculate the baseline prevalence of inadequate Ca intake using the corresponding EAR and UL for Ca defined by IOM for each population group. Prevalence of low Ca intake was calculated as the proportion of individuals in the group with usual Ca intake below the age-specific EAR, and prevalence of excess was calculated as the proportion of individuals with usual Ca intakes above the age-specific UL^(32)^.

After calculating the prevalence of Ca intake inadequacy for each population group, we selected the group with the highest prevalence of low Ca intake and defined a desired ‘target prevalence of inadequate intake’ to plan the water fortification simulation.

We then calculated the initial gap defined as the estimated amount of Ca that should be added to water in order to achieve the desired ‘target prevalence of inadequate intakes’. The initial gap was calculated as difference in mg/d between the desired prevalence of that group.

Subsequently, using IMAPP, we estimated the Ca intake distributions after simulating the addition of Ca to each individual water intake. In the case that the dietary information did not contain the amount of water intake, we simulated two scenarios: the consumption of 1 and then 1·5 litres of water intake per day for individuals older than 1 year and 0·6 and 0·8 litres of water intake per day for infants aged 6 to <12 months. We first simulated adding 500 mg of elemental Ca/l of water. We selected 500 mg of Ca as it is the approximate gap between Ca intake in LMIC and HIC^(5,12)^. In cases where the percentage of individuals exceeded the upper limit above 2·5 % in any of the groups, we decreased the amount of Ca to 400, 300 or 200 mg/l of water. We then re-calculated the prevalence of Ca inadequacy of each population group in the database after simulating the addition of different amounts of Ca to water.

## Results

The number of participants for each age group, prevalence of low Ca intake and percentage of individuals exceeding the upper limit as well as the changes on Ca intake after simulating the addition of Ca to water for each country are described in Tables 1–7. The only databases with information on water intake were those from the USA and Italy, and for the rest of the countries, Uganda, Lao PDR, Bangladesh, Zambia, Italy and Argentina, we simulated an intake of 1 and 1·5 litres of water/individual per d.

**Table 1:** Simulation of the effect of water fortification with 500 mg of calcium/l on calcium intake (Uganda)

Group	*n*	EAR (mg)	Basal Ca intake				Ca intake after simulation of intake of 1 litre of fortified water a day				Ca intake after simulation of intake of 1·5 litre of fortified water a day			
			Mean (mg)	sd	<EAR (%)	>UL (%)	Mean (mg)	sd	<EAR (%)	>UL (%)	Mean (mg)	sd	<EAR (%)	>UL (%)
Women non-pregnant														
19 ≤ age < 31	69	800	462·7	283·7	89·8	0·0	958·9	283·0	31·0	0·3	1209·6	277·2	0·0	0·5
31 ≤ age < 51	171	800	363·3	163·2	98·1	0·0	860·3	160·8	40·8	0·0	1110·4	167·3	0·2	0·0
51 ≤ age < 71	30	1000	418·9	220·3	98·0	0·0	919·4	218·2	70·8	0·1	1169·2	216·9	22·1	0·4
Pregnant women														
19 ≤ age < 31	172	800	372·3	118·5	99·6	0·0	897·6	206·0	37·2	0·0	1148·4	209·5	0·0	0·0
31 ≤ age < 51	123	800	389·3	137·4	99·0	0·0	887·1	158·5	32·7	0·0	1139·4	161·0	0·0	0·0

EAR, estimated average requirement; UL, upper limit.

**Table 7:** Simulation of the effect of water fortification with 500 mg of calcium/l on calcium intake (USA)

Group	*n*	EAR (mg)	Water Intake	Basal Ca intake				Ca intake after fortification of water with 500 mg of Ca/l				Ca intake after fortification of water with 400 mg of Ca/l				Ca intake after fortification of water with 300 mg of Ca/l				Ca intake after fortification of water with 200 mg of Ca/l			
				Mean (mg)	sd	<EAR (%)	>UL (%)	Mean (mg)	sd	<EAR (%)	>UL (%)	Mean (mg)	sd	<EAR (%)	>UL (%)	Mean (mg)	sd	<EAR (%)	>UL (%)	Mean (mg)	sd	<EAR (%)	>UL (%)
Children																							
0 ≤ age < 1	222	270	44·5	650·0	288·4	3·7	1·7	671·7	299·1	3·3	2·1	667·4	296·4	3·3	2·0	663·0	294·0	3·4	1·9	658·7	291·9	3·5	1·9
1 ≤ age < 4	564	400	289·3	915·0	492·0	11·1	1·1	1059·0	535·3	6·5	2·0	1030·2	522·5	7·0	1·7	1001·4	511·6	7·7	1·5	972·6	502·8	8·6	1·3
4 ≤ age < 9	828	640	438·6	952·3	487·6	28·9	0·7	1171·4	556·7	16·7	2·2	1127·6	535·8	18·2	1·7	1083·8	518·1	20·1	1·3	1040·0	504·0	22·5	1·0
Women non-pregnant																							
9 ≤ age < 14	417	1100	539·7	919·1	547·9	69·2	0·4	1188·8	643·9	51·0	1·3	1134·8	615·2	54·3	1·0	1080·9	590·7	58·0	0·7	1027·0	570·9	61·8	0·6
14 ≤ age < 19	383	1100	775·2	826·8	520·9	75·4	0·3	1214·2	731·9	51·7	2·6	1136·7	674·1	55·7	1·7	1059·2	622·2	60·3	1·0	981·7	577·8	65·3	0·6
19 ≤ age < 31	511	800	1242·5	825·9	501·5	56·3	0·9	1446·8	843·4	23·7	11·2	1322·6	750·7	26·8	7·5	1198·5	666·2	31·0	4·5	1074·3	593·0	37·0	2·4
31 ≤ age < 51	854	800	1236·1	837·7	471·8	54·6	0·6	1455·3	786·9	21·0	10·2	1331·8	701·9	24·1	6·6	1208·3	624·0	28·3	3·7	1084·8	556·7	34·5	1·9
51 ≤ age < 71	845	1000	1166·5	775·7	436·4	74·9	1·6	1358·5	736·0	35·8	17·6	1242·0	655·8	40·9	12·6	1125·4	582·2	47·4	8·1	1008·8	518·1	55·7	4·7
71 ≤ age	381	1000	899·5	731·7	418·3	78·6	1·3	1180·7	627·6	44·2	10·0	1091·0	567·1	49·9	7·0	1001·1	512·4	56·6	4·5	911·3	467·2	64·2	2·8
Pregnant women																							
19 ≤ age < 31	37	800	1574·4	999·0	542·3	40·5	1·2	1773·4	879·4	13·5	20·1	1618·4	777·4	15·2	13·3	1463·5	687·0	17·6	7·3	1308·6	612·7	21·6	3·3
31 ≤ age < 51	26	800	1481·2	947·7	507·5	42·8	0·6	1690·1	1136·1	23·2	20·7	1541·4	978·3	24·4	15·7	1392·4	825·6	26·2	10·1	1244·2	684·5	28·4	4·8
Male																							
9 ≤ age < 14	412	1100	579·2	1039·3	650·0	62·2	1·3	1328·1	760·8	45·2	3·5	1270·4	728·2	48·1	2·8	1212·6	700·7	51·5	2·3	1154·8	678·1	55·1	1·9
14 ≤ age < 19	374	1100	859·2	1100·3	698·9	58·1	1·8	1529·1	963·5	38·9	7·9	1443·3	892·9	41·6	6·1	1357·6	828·7	44·9	4·6	1271·8	773·2	48·7	3·3
19 ≤ age < 31	497	800	1367·6	1067·7	719·6	42·9	4·7	1751·2	1132·4	19·9	21·4	1614·6	895	1023·6	22·0	1477·9	924·0	24·9	13·1	1341·3	835·0	29·0	9·4
31 ≤ age < 51	792	800	1318·9	1042·1	658·1	42·7	3·5	1701·4	988·3	17·6	18·8	1569·6	895·2	19·7	14·4	1437·8	811·8	22·8	10·4	1305·9	741·4	27·2	7·2
51 ≤ age < 71	824	800	1054·9	939·1	581·7	47·9	5·8	1465·9	900·1	22·9	21·7	1360·6	814·3	25·4	17·6	1255·2	736·4	28·8	13·7	1149·8	669·1	33·5	10·2
71 ≤ age	372	1000	779·4	871·6	553·8	67·1	3·9	1260·6	745·7	42·3	14·5	1182·5	689·1	46·0	11·5	1105·1	645·2	48·4	7·8	1027·3	604·0	53·8	5·9

EAR, estimated average requirement; UL, upper limit.

We calculated the usual Ca intake with the information from repeated measures except for Argentina, as the database had only one recall and for Lao and pregnant women of Italy, where the number of cases with duplicate recalls where small. In these cases, we used the 2003–2008 NHANES coefficient of variance provided by IMAPP as the external variance.

Below we report the results of the mean Ca intake, prevalence of low Ca intake and percentage of Ca intake above the upper limit for each of the IOM age groups before and after fortification.

### Uganda

The mean daily Ca intake ranged from 363·3 (sd 163·2) to 462·7 (sd 283·7) mg in non-pregnant women and from 372·3 (sd 118·5) to 389·3 (sd 137·4) mg in pregnant women. The prevalence of low Ca intake was approximately 90 % or higher in all population groups. None of the population age groups had Ca intake exceeding the recommended upper limits for Ca (Table 1).

After simulating the intake of water fortified with 500 mg of Ca /l assuming an intake of 1 litre of water/d, the prevalence of low Ca intake decreased to 31 % in non-pregnant women aged 19 to <31 years, to 40·8 % in non-pregnant women aged 31 to <51 years and to 70·8 % in non-pregnant women aged 51 to <71 years (Table 1). After simulating the intake of water fortified with 500 mg of Ca/l assuming an intake of 1·5 litres of water/d, the prevalence of low Ca intake decreased to 0 % in women aged 19 to <<51 years and to 22·1 % in the group aged 51 to <<71 years. None of the groups had more than 0·5 % of the individuals with Ca intake exceeding the recommended upper limit for Ca.

The original distribution of Ca intake and distribution of Ca intake after the simulation of water fortification with 500 mg of Ca/l assuming an intake of 1 litre of water/d are shown in online supplementary material, Supplemental Fig. S1.

### Lao People’s Democratic Republic

The mean daily Ca intake ranged from 214·1 (sd 104·6) to 364·1 (sd 257·8) mg in children, from 207·2 (sd 93·4) to 263·5 (sd 109·9) mg in girls and non-pregnant women, from 229·6 (sd 103·1) to 313·7 (sd 185·5) mg in pregnant women and from 212·0 (sd 62·5) to 435·6 (sd 318·3) mg in men. The prevalence of low Ca intake ranged from 58·4 to 99·5 % in children and it was around 90 % in the rest of the groups, and none of them had Ca intake higher than 2 % of the upper limit (Table 2).

**Table 2:** Simulation of the effect of water fortification with 500 mg of calcium/l on calcium intake (Lao PDR)

Group	*n*	EAR (mg)	Basal Ca intake				Ca intake after simulation of intake of 1 litre of fortified water a day				Ca intake after simulation of intake of 1·5 litre of fortified water a day			
			Mean (mg)	sd	<EAR (%)	>UL (%)	Mean (mg)	sd	<EAR (%)	>UL (%)	Mean (mg)	sd	<EAR (%)	>UL (%)
Children														
0·5 ≤ age < 1	170	270	328·8	398·6	62·3	2·0	629·0*	371·9	0·0	3·2	728·7†	363·7	0·0	3·8
1 ≤ age < 4	407	400	364·1	257·9	66·8	0·0	866·9	267·9	0·0	0·0	1116·7	267·3	0·0	0·2
4 ≤ age < 9	294	640	214·1	104·6	99·5	0·0	713·0	100·4	24·8	0·0	962·4	99·6	0·0	0·0
Women non-pregnant														
9 ≤ age < 14	74	1100	207·2	93·4	100·0	0·0	704·6	83·9	99·9	0·0	954·9	83·8	93·5	0·0
14 ≤ age < 19	38	1100	207·7	166·8	99·5	0·0	702·7	146·6	97·6	0·0	951·8	143·5	88·8	0·0
19 ≤ age < 31	53	800	263·5	109·9	99·8	0·0	761·5	108·6	71·6	0·0	1010·7	107·6	0·0	0·0
31 ≤ age < 51	101	800	250·6	105·4	99·8	0·0	749·9	102·3	75·7	0·0	999·9	101·9	0·0	0·0
51 ≤ age < 71	127	1000	230·0	125·3	99·9	0·0	736·8	138·9	94·9	0·0	990·3	148·2	63·4	0·1
Pregnant women														
14 ≤ age < 19	23	1100	229·6	103·1	100·0	0·0	754·6	91·7	99·8	0·0	1004·7	91·6	85·3	0·0
19 ≤ age < 31	196	800	313·7	185·5	97·6	0·0	820·7	189·7	55·7	0·0	1067·7	186·3	0·2	0·0
31 ≤ age < 51	66	800	300·6	167·1	98·3	0·0	798·1	151·8	60·0	0·0	1046·8	149·3	0·0	0·0
Male														
19 ≤ age < 31	22	800	212·0	62·5	100·0	0·0	712·2	63·2	90·8	0·0	962·3	63·6	0·0	0·0
31 ≤ age < 51	108	800	435·6	381·3	89·7	0·5	903·2	283·0	45·0	0·3	1149·3	274·1	0·0	0·5
51 ≤ age < 71	116	800	276·1	137·8	99·3	0·0	777·6	141·6	67·1	0·0	1027·9	141·5	0·1	0·0
71 ≤ age	21	1000	342·4	222·0	98·1	0·1	904·47	313·0	75·8	1·4	1098·9	236·8	39·5	1·0

EAR, estimated average requirement; UL, upper limit.

* Assuming 600 ml of water intake/d.

† Assuming 800 ml of water intake/d.

After simulating the intake of water fortified with 500 mg of Ca/l assuming an intake of 1 litre of water/d, the prevalence of low Ca intake decreased to 0 % in children <4 years, to 24·8 in children aged 4 to <9 years, to between 45·0 and 90·8 % in men aged 19 and over, to between 55·7 and 99·8 % in pregnant women aged 14 to <51 years and to between 71·6 and 99·9 % in women aged 14 to <71 years. (Table 2) None of the groups had more than 1·5 % of the individuals with Ca intake exceeding the recommended upper limit for Ca, except for infants 6–12 months were the percentage exceeding the UL was 3·2 %.

The original distribution of Ca intake and the distribution of Ca intake after the simulation of water fortification with 500 mg of Ca/l of water assuming an intake of 1 litre of water/d are shown in online supplementary material, Supplemental Fig. S2.

### Bangladesh

The mean daily Ca intake ranged from 151·8 (sd 53·7) to 160·8 (sd 55·6) mg in non-pregnant women and from 143·2 (sd 80·3) to 150·2 (sd 58·8) mg in pregnant women. The prevalence of low Ca intake was 100 % in all age groups. None of the groups had individuals with a Ca intake exceeding the recommended upper limit for Ca (Table 3).

**Table 3:** Simulation of the effect of water fortification with 500 mg of calcium/l on calcium intake (Bangladesh)

Group	*n*	EAR (mg)	Basal Ca intake				Ca intake after simulation of intake of 1 litre of fortified water a day				Ca intake after simulation of intake of 1·5 litre of fortified water a day			
			Mean (mg)	sd	<EAR (%)	>UL (%)	Mean (mg)	sd	<EAR (%)	>UL (%)	Mean (mg)	sd	<EAR (%)	>UL (%)
Women non-pregnant														
19 ≤ age < 31	157	800	160·8	55·6	100·0	0·0	660·5	56·1	97·6	0·0	910·9	55·9	0·0	0·0
31 ≤ age < 51	67	800	151·8	53·7	100·0	0·0	650·8	55·0	98·3	0·0	901·4	55·3	0·2	0·0
Pregnant women														
19 ≤ age < 31	174	800	150·2	58·8	100·0	0·0	653·1	70·8	95·8	0·0	903·5	71·2	0·0	0·0
31 ≤ age < 51	62	800	143·2	80·3	100·0	0·0	635·5	69·2	96·9	0·0	885·9	69·2	0·8	0·0

EAR, estimated average requirement; UL, upper limit.

After simulating the intake of water fortified with 500 mg of Ca/l and assuming an intake of 1 litre of water/d, the prevalence of low Ca intake decreased to around 95 % in all age groups (Table 3). After simulation, the intake of water fortified with 500 mg of Ca/l and assuming an intake of 1·5 litres of water/d, the prevalence of low Ca intake decreased to nearly 0 % in all age groups. None of the groups had more than 0·5 % of the individuals with Ca intake exceeding the recommended upper limit for Ca.

The original distribution of Ca intake and the distribution of Ca intake after the simulation of water fortification with 500 mg of Ca/l of water assuming an intake of 1 litre of water/d are shown in online supplementary material, Supplemental Fig. S3.

### Zambia

The mean daily Ca intake ranged from 202·6 (sd 70·8) to 220·2 (sd 64·0) mg in children, from 311·5 (sd 24·5) to 339·3 (sd 9·6) mg in non-pregnant women and from 317·4 (sd 85·6) to 322·2 (sd 114·4) mg in pregnant women. The prevalence of low Ca intake was 98 % or higher in all population groups. None of the groups had individuals with Ca intakes exceeding the recommended upper limit for Ca (Table 4).

**Table 4:** Simulation of the effect of water fortification with 500 mg of calcium/l on calcium intake (Zambia)

Group	*n*	EAR (mg)	Basal Ca intake				Ca intake after simulation of intake of 1 litre of fortified water a day				Ca intake after simulation of intake of 1·5 litre of fortified water a day			
			Mean (mg)	sd	<EAR (%)	>UL (%)	Mean (mg)	sd	<EAR (%)	>UL (%)	Mean (mg)	sd	<EAR (%)	>UL (%)
Children														
1 ≤ age < 4	322	400	202·6	70·8	98·7	0·0	702·6	71·0	0·0	0·0	952·7	70·8	0·0	0·0
4 ≤ age < 9	132	640	220·2	64·0	100·0	0·0	720·6	62·0	6·9	0·0	970·9	61·8	0·0	0·0
Women non-pregnant														
19 ≤ age < 31	73	800	339·3	9·6	100·0	0·0	840·0	6·1	0·0	0·0	1089·3	7·8	0·0	0·0
31 ≤ age < 51	72	800	311·5	24·5	100·0	0·0	811·5	33·4	38·3	0·0	1061·8	35·6	0·0	0·0
Pregnant women														
19 ≤ age < 31	126	800	322·2	114·4	99·8	0·0	821·0	109·6	47·6	0·0	1071·0	108·8	0·0	0·0
31 ≤ age < 51	60	800	317·4	85·6	100·0	0·0	817·0	88·8	45·4	0·0	1067·1	89·2	0·0	0·0

EAR, estimated average requirement; UL, upper limit.

After the simulation of water fortification with 500 mg/l assuming an intake of 1 litre of water/d, the prevalence of low Ca intake decreased to <50 % in all age groups (Table 4). After the simulation of water fortified with 500 mg of Ca/l and assuming an intake of 1·5 litres of water/d, the prevalence of low Ca intake decreased to 0 % in all age groups. None of the groups had individuals with Ca intakes exceeding the upper limit.

The original distribution of Ca intake and the distribution of Ca intake after water fortification with 500 mg of Ca/l of water assuming an intake of 1 litre of water/d are shown in online supplementary material, Supplemental Fig. S4.

### Argentina

The mean daily Ca intake ranged from 609·3 (sd 348·0) to 752·2 (sd 323·4) mg in children, from 361·6 (sd 180·2) to 460·7 (sd 167·6) mg in non-pregnant women and from 470·6 (sd 246·7) to 491·3 (sd 244·7) in pregnant women. The prevalence of low Ca intake was 17·5 % in infants <1 year, 13·4 % in children aged 1 to <4 years, 46·2 % in children aged 4 to <9 years and 88 % or higher in girls and women, including pregnant women. None of the groups had individuals with a Ca intake exceeding the recommended upper limit for Ca, except for infants <1 year (Table 5).

**Table 5:** Simulation of the effect of water fortification with 500 mg of calcium/l on calcium intake (Argentina)

Group	*n*	EAR (mg)	Basal Ca intake				Ca intake after simulation of intake of 1 litre of fortified water a day				Ca intake after simulation of intake of 1·5 litre of fortified water a day			
			Mean (mg)	sd	<EAR (%)	>UL (%)	Mean (mg)	sd	<EAR (%)	>UL (%)	Mean (mg)	sd	<EAR (%)	>UL (%)
Children														
0·5 ≤ age < 1	2110	270	609·3	348·0	17·5	1·5	909·6*	348·2	0·0	6·1	1009·6†	348·1	0·0	9·3
1 ≤ age < 4	7787	400	752·2	323·4	13·4	0·0	1252·5	324·3	0·0	0·1	1502·5	324·3	0·0	0·5
4 ≤ age < 9	3480	640	683·3	260·6	46·2	0·0	1182·5	258·3	0·4	0·0	1432·5	258·3	0·0	0·0
Women non-pregnant														
9 ≤ age < 14	864	1100	460·7	167·6	99·9	0·0	938·1	197·8	80·6	0·0	1188·1	197·7	27·6	0·0
14 ≤ age < 19	1122	1100	438·8	198·2	99·4	0·0	902·0	168·1	81·0	0·0	1152·0	167·9	37·7	0·0
19 ≤ age < 31	2112	800	402·6	168·1	97·6	0·0	862·1	180·0	30·4	0·0	1112·4	180·1	0·0	0·0
31 ≤ age < 51	2507	800	361·6	180·2	97·6	0·0	960·4	166·8	42·7	0·0	1210·5	166·7	0·0	0·0
Pregnant women														
14 ≤ age < 19	197	1100	470·6	246·7	97·8	0·0	970·8	248·0	75·6	0·0	1219·8	244·9	35·9	0·0
19 ≤ age < 31	963	800	490·5	240·1	89·0	0·0	990·5	240·0	23·1	0·0	1240·5	239·9	0·0	0·0
31 ≤ age < 51	450	800	491·3	244·7	88·4	0·0	991·3	245·3	24·2	0·0	1241·4	245·4	0·0	0·0

EAR, estimated average requirement; UL, upper limit.

* Assuming 600 ml of water intake/d.

† Assuming 800 ml of water intake/d.

After the simulation of water fortification with 500 mg/l assuming an intake of 1 litre of water/d, the prevalence of low Ca intake decreased to 0 % in children <4 years, 0·4 % in children aged 4 to <9 years. The prevalence of low Ca intake was reduced to around 80 % in girls aged 9 to <19 years, to 30·4 % in women aged 19 to <31 and to 42·7 % in women aged 31 to <51 years. In pregnant women, the prevalence of low Ca intake was reduced to 75·6 % in those aged 14 to <19 years, to 23·1 % in those aged 19 to <31 years and to 24·2 % in those aged 31 to <51 years (Table 5). None of the groups had more than 0·5 % of the individuals with Ca intakes exceeding the upper limit, except for infants aged 6 months to <1 year that had 6·1 %.

After the simulation of water fortified with 500 mg of Ca/l and assuming an intake of 1·5 litres of water/d, the prevalence of low Ca intake decreased to nearly 0 % in most age groups except for girls aged 9 to <14 years (27·6 %), for girls 14 to <19 years (37·7 %) and for pregnant girls aged 14–19 years (35·9 %). None of the groups had more than 0·5 % of the individuals with Ca intakes exceeding the upper limit, except for infants aged 6 months to <1 year that had 9·3 %.

The original distribution of Ca intake and the distribution of Ca intake after water fortification with 500 mg of Ca/l of water simulating the intake of 1 litre of water/d are shown in online Supplemental Fig. S5.

### Italy

The mean daily Ca intake ranged from 577·0 (sd 127·5) to 728·6 (sd 180·5) mg in children, from 822·0 (sd 267·6) to 1009·1 (sd 181·2) mg in pregnant women, from 716·4 (237·5) to 819·5 (sd 187·0) mg in girls and non-pregnant women and from 790·2 (sd 257·8) to 917·2 (sd 275·0) mg in boys and men. The prevalence of low Ca intake ranged from 0·3 to 37·2 % in children, from 13·1 to 51·0 % in pregnant women, from 65·7 to 92·3 in girls and non-pregnant women and from 44·3 to 82·8 % in boys and men. None of the groups had >0·2 % of the individuals with Ca intakes exceeding the recommended upper limit for Ca (Table 6).

**Table 6:** Simulation of the effect of water fortification with 500 mg of calcium/l on calcium intake (Italy)

Group	*n*	EAR (mg)	Water Intake	Basal Ca intake				Ca intake after fortification of water with 500 mg of Ca/l				Ca intake after fortification of water with 400 mg of Ca/l			
				Mean (mg)	sd	<EAR (%)	>UL (%)	Mean (mg)	sd	<EAR (%)	>UL (%)	Mean (mg)	sd	<EAR (%)	>UL (%)
Children															
0 ≤ age < 1	13	270	444·0	577·0	127·5	0·3	0·0	790·6	205·8	0·0	0·0	747·5	182·4	0·0	0·0
1 ≤ age < 4	53	400	415·3	728·6	180·5	1·9	0·0	930·2	221·1	0·1	0·0	889·9	211·4	0·2	0·0
4 ≤ age < 9	145	640	513·6	725·9	210·6	37·2	0·0	974·6	255·0	6·6	0·0	924·8	244·2	9·6	0·0
Women pregnant															
19 ≤ age < 31	4	800	1146·9	1009·1	181·2	13·1	0·0	1363·2	134·9	0·0	0·0	1355·8	121·1	0·0	0·0
31 ≤ age < 51	24	800	823·6	822·0	267·6	51·0	0·0	1257·1	374·1	9·4	0·4	1169·9	350·3	13·6	0·2
Women non-pregnant															
9 ≤ age < 14	83	1100	592·1	791·2	247·8	88·9	0·0	1090·7	316·1	57·2	0·0	1030·9	299·4	64·7	0·0
14 ≤ age < 19	84	1100	662·1	819·5	187·0	92·3	0·0	1151·1	270·3	46·6	0·0	1084·5	248·9	56·7	0·0
19 ≤ age < 31	261	800	789·8	716·4	237·5	68·4	0·0	1105·0	340·2	18·6	0·1	1027·1	314·1	24·8	0·0
31 ≤ age < 51	551	800	683·1	734·9	236·3	65·7	0·0	1078·3	309·5	17·8	0·0	1009·6	287·9	23·8	0·0
51 ≤ age < 71	482	1000	695·7	756·0	243·9	84·7	0·0	1103·4	323·8	41·0	1·0	1033·8	303·2	49·7	0·5
71 ≤ age	206	1000	661·8	799·7	228·4	81·8	0·0	1141·9	306·7	34·6	0·8	1073·5	287·7	43·1	0·4
Male															
9 ≤ age < 14	83	1100	627·5	881·9	325·0	78·0	0·0	1189·7	383·8	45·5	0·0	1128·1	369·7	52·3	0·0
14 ≤ age < 19	68	1100	754·6	835·3	297·9	82·8	0·0	1205·0	376·0	43·7	0·0	1130·8	355·9	52·0	0·0
19 ≤ age < 31	208	800	780·0	859·4	239·3	44·3	0·0	1239·2	343·3	7·8	0·3	1163·4	317·5	11·0	0·1
31 ≤ age < 51	481	800	685·7	790·2	257·8	56·8	0·0	1132·9	363·5	17·7	0·2	1064·2	337·8	22·5	0·1
51 ≤ age < 71	418	800	692·3	838·7	283·7	49·6	0·2	1182·1	366·3	13·8	2·6	1113·5	344·7	18·0	1·5
71 ≤ age	105	1000	643·8	917·2	275·0	66·2	0·2	1243·7	376·3	27·8	3·9	1178·5	354·9	34·0	2·5

EAR, estimated average requirement; UL, upper limit.

After the simulation of water fortification with 500 mg/l taking into account the reported water intake of each individual, the prevalence of low Ca intake decreased to <57 % in all age groups; however, the percentage of individuals exceeding the upper limit increased to between 2·6 and 3·9 % in men older than 51 years (Table 6). We then simulated a fortification with lower Ca concentrations. With 400 mg of Ca/l of water, we found that the prevalence of inadequate Ca intake reached 64·7 % in some age groups like boys and girls aged 9 to <19 years. On the other, the percentage of individuals exceeding the upper limit decreased to 1·5 % in men aged 31 to <51 years and to 2·5 % in men older than 51 years.

The original distribution of Ca intake and the distribution of Ca intake after water fortification are shown in online supplementary material, Supplemental Fig. S6.

### USA

The mean daily Ca intake ranged from 650·0 (sd 288·4) to 952·3 (sd 487·6) mg in children, from 731·7 (sd 418·3) to 919·1 (sd 547·9) mg in girls and non-pregnant women, from 947·7 (sd 507·5) to 999·0 (sd 542·3) mg in pregnant women and from 871·6 (sd 553·8) to 1100·3 (sd 698·9) mg in boys and men. The prevalence of low Ca intake ranged from 3·7 to 28·9 % in children, from 54·6 to 78·6 % in girls and non-pregnant women, from 40·5 to 42·8 % in pregnant women and from 42·9 to 67·1 % in boys and men. Children and women did not exceed the Ca intake upper limit in >2 %, whereas around 3·5–5·8 % of the individuals exceeded the upper limit in the group of men older than 31 years (Table 7).

After the simulation of water fortification with 500 mg/l and taking into account the reported water intake of each individual, the prevalence of low Ca intake decreased in all age groups; however, the percentage of individuals exceeding the upper limit increased to 20 % in pregnant women and between 14·5 and 21·7 % in males older than 19 years (Table 7). We then simulated a fortification with 400 and 300 mg of Ca/l of water and found that pregnant women and young men groups still had around 10 % of individuals exceeding the upper limit. Finally, we simulated the fortification with 200 mg of Ca/l of water and found that the prevalence of inadequate intake was still high in men aged 51 to <71 years; however, the percentage of individuals exceeding the upper limit decreased to 10 % or less.

The original distribution of Ca intake and the distribution of Ca intake after simulating water fortification are shown in online Supplemental Fig. S7.

**Fig. 1:**
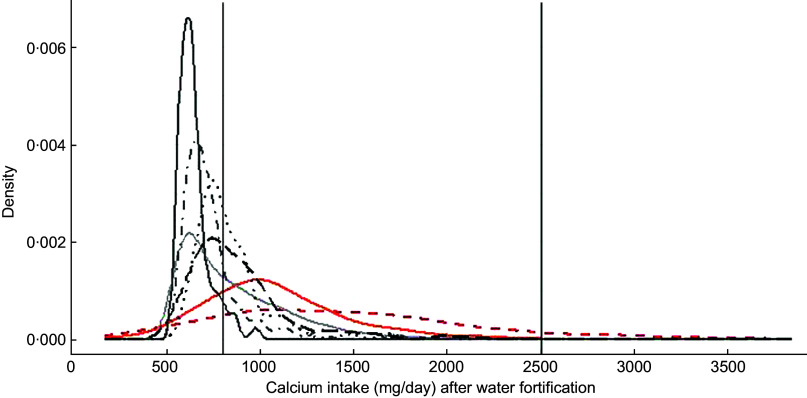
Simulation of water fortification. Country: 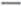, Argentina; 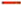, Italy; 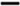, Uganda; 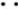, Zambia; 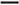, Bangladesh; 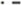, Lao; 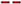, USA

Finally, Fig. 1 presents the distribution of Ca intake of pregnant women aged between 31 and 50·9 years after simulating the fortification of water with 500 mg of Ca/l. For Argentina, Lao, Uganda, Zambia and Bangladesh, the simulation assumes that each woman drinks 1 litre of water/d, whereas for Italy and USA, the simulation takes into account the individual water intake. The figure shows that after water fortification, most of the population of HIC had Ca intake between 800 and 2500 mg of Ca a day which are the limits considered safe for Ca, whereas all LMIC had most of their populations under 800 mg of Ca/d.

## Discussion

The current study analyses the adequacy of Ca intake in populations from Uganda, Zambia, Bangladesh, Lao PDR, Argentina, Italy and USA. It describes the usual Ca intake, the prevalence of low Ca intake and the percentage of individuals with Ca intake above the upper limit using the IMAPP developed by IOWA. We then described the changes in the Ca intake distribution for each IOM age group after the simulation of a water fortification strategy.

In the current study, we found that for all the dietary databases assessed from LMIC, the strategy of fortifying water with 500 mg of Ca/l would decrease the prevalence of low Ca intake in all age groups. We also found that this strategy in the LMIC assessed would be safe as no group would present a percentage of individuals exceeding the upper limit in more than 2 %. In Italy, a strategy of fortifying water with 500 mg of Ca/l would lead to between 2·6 and 3·9 % of men aged 31 years or more exceeding the upper limit. We simulated fortifying water with 400 mg of Ca/l and found that although it would decrease the prevalence of low Ca intake, it would leave 2·5 % of men aged 51 years or older exceeding the upper limit for Ca intake.

On the other hand, in the USA, even though there were groups with high prevalence of low Ca intake, a strategy to fortify water with Ca would lead to safety issues. In the USA, water fortified with 200 mg of Ca/l would lead to a decrease in the prevalence of low Ca intake; however, some groups would have exceeded the upper limit in around 10 %. Our analysis shows that in countries like the USA, even when the water intake is lower than recommended, a population-based policy to fortify water would not be recommended. Strategies to increase Ca should focus on specific population groups with low Ca intake.

The current study uses information from different countries with diverse age groups including pregnant women, which gives strength to the results. Most data were collected using duplicate 24 h recalls or food records that are validated methodologies to measure Ca intake^(33)^. Only one country, Argentina, had a single 24 h recall that as recommended was adjusted with external variability to obtain estimates of Ca inadequacy, the complex sampling of this database was also taken into account in the analysis^(34)^.

One limitation of the current study is that dietary surveys from Uganda, Lao PDR, Bangladesh, Zambia and Argentina did not collect water intake for each individual in the sample. In this way, if actual water intake was less than the simulated 1 litre/d, the reduction of Ca intake inadequacy would be lower than the one shown here. Furthermore, as water was not registered in the surveys, the basal Ca provided by local water intake was not calculated. We did not find data on the Ca concentration of water from Uganda, Lao PDR, Bangladesh or Zambia to estimate if this could impact the total Ca intake estimated with these databases. In Argentina, there is evidence from pregnant women that water could contribute to around 6 % of total Ca intake; however, due to the large prevalence of low Ca intake, this information would not change the results presented in the current study^(35)^.

Another limitation of the current study is that we were not able to assess the across person variability of water intake for most of the countries as water consumption was simulated for each individual in these databases; thus, the real distribution of Ca intake would probably be different from the one presented here. However, although we did not know the exact amount of water intake, water is generally consumed by all individuals and on a daily basis^(36)^. A further limitation of the current study is the small sample size in some age groups so for those specific groups the results should be interpreted with caution.

We calculated the percentage of individuals above the upper limit of Ca intake for infants <1 year taking into account recommendations of water intake as it is unlikely that they consume 1 litre of water a day, especially those under 6 months were the recommendation is exclusive breastfeeding^(37)^. Recommendations of water intake from beverages, excluding water from foods, are 600 to 800 ml/d for infants aged 6 to <12 months, 900 ml to 1 litre for children aged 1 to <4 years, 1·2 litres for children aged 4 to <9 years, 1·5–1·8 litres/d for older children and 1·5–2 litres/d for adults and pregnant women^(38,39)^. Taking into account the USA data analysed in the current study, daily water intake seems to be lower than the recommended amounts^(29)^. Studies from Britain, Spain and Italy also show that water intake is lower than the recommended^(40,41)^.

Water intake information is not always collected in dietary surveys as shown by the analysis of these databases. For this article, we searched national or subnational databases with information of daily food and nutrient obtained from 24 h recalls or daily food records. Of the databases analysed, only two had data on water intake. These two databases were from HIC and none from LMIC. The lack of data on water intake and Ca concentration of water in the populations of LMIC included in the current study are the main limitations of this study. Also, there is limited information on the type of drinking water as many dietary surveys do not collect these data in detail^(40)^. Water intake is difficult to estimate, and there is no standardised form to collect these data. Information on the amount and source of water consumed would be required to better tailor a water fortification strategy for a particular population.

Although, the current study does not take in account special groups with extreme water intake that could be put at risk of excess Ca intake with a water fortification strategy, we found that even in HIC, very few people consumed more than 1·5 litres of water a day. In Italy, only 4·4 % (56/1280) of men and 4·3 % (69/1612) of women aged 14 years or more had intakes higher than 1·5 litres of water. In USA, 26·8 % (767/2859) of men and 27·4 % (832/3037) of women aged 14 years or higher had intakes higher than 1·5 litres of water.

Studies have refuted some Ca supplementation side effects like detriment of iron status, formation of renal stones and myocardial infarction in older people. However, in some supplementation studies, a few deleterious effects, such as postpartum bone resorption and gastrointestinal discomfort, have been reported.

One study showed that the consumption of Ca-rich water can provide a quarter of total Ca daily intake and it can be a recommendation to increase Ca intake in people with low intake of dairy products^(42)^. Another study from Poland showed that tap water with around 68–114 mg of Ca/l can contribute to 6–14 % of total Ca intake of young women. Finally, a study from the UK showed that Ca intake in areas where Ca concentration of water reaches 300 mg of Ca/l can contribute to 8 % of adolescents total Ca intake^(43,44)^.

Mineral waters with high concentration of Ca, around 300 mg/l or more, are found in Italy, Spain, the UK and France^(44–46)^. Ca concentration of artificially mineral bottled waters is much lower, and technical research to increase Ca content of artificially mineral and or tap water is required to improve Ca intake of low Ca intake populations from areas where local water has low Ca concentrations^(47)^.

WHO recommends a minimum of 20 mg of Ca/l of water and to prevent risk of CVD suggest an optimal concentration between 50 and 80 mg/l^(48)^. In this way, Ca concentration of water should be regulated more tightly as it could be a good source of minerals for the diet. Regulations should control a minimum level of Ca in tap water so as to improve health, specially taking into account that waters with low Ca and magnesium could be corrosive and harmful^(49)^.

Currently, tap water regulations include a maximum level of Ca concentration, while fewer include a minimum Ca concentration, which if so they usually focus on hardness. Furthermore, Ca concentration values are usually provided for technical rather than health considerations^(48)^. In some US states, the minimum Ca concentration required is 10 mg/l; however, based on health concerns, WHO recommends Ca concentrations of no <20–30 mg/l with an optimal amount of 40–80 mg/l^(49–51)^. We have reviewed international and national food and beverage regulations, and the policy to add Ca to water is present only in those areas with demineralised and/or desalinated water^(48)^. Our results show that Ca in water can improve the diet of populations with low Ca intake and thus we consider that setting a minimum Ca concentration level in water could contribute to attain an adequate Ca intake and its health benefits^(52)^ help to improve the health of those populations with low Ca intake.

After these preliminary results, if water fortification is feasible, the implementation of this policy would require in each particular population the assessments of infrastructure, water sources, funds, local regulations and acceptability (such as change in taste) according to the selected fortification process.

## Conclusion

We found that for most LMIC countries, increasing Ca concentration of water to 500 mg of Ca/l would decrease the prevalence of Ca intake inadequacy without exceeding the upper level of Ca intake in any population group. On the other hand, if the water intake is 1·5 litres a day, the amount of Ca would still be safe in LMIC, except for women aged 19–31 years in Lao PDR.

The current study confirms that low Ca intake can also be found within some population age groups of HIC. In USA, Ca intake has a widespread distribution, implying that some individuals have very low or very high intakes within the same age group. In similar situations where Ca intake levels are extreme such as in the USA, a strategy reaching those specific groups should be put in place.

Food fortification is the most cost-effective intervention to improve micronutrient intake; however, a comprehensive evaluation of its impact should include a simulation analysis like the one presented.

The current study reinforces the need to plan Ca fortification strategies given the health relevance of an adequate Ca intake and the high percentage of individuals in LMIC who fail to meet the requirements. Water is an interesting vehicle because it is of universal and equitable intake showing a good bioavailability that does not imply an increase in energy intake that can contribute to obesity. Furthermore, unlike other foods, no population group has dietary restrictions to water.

Fortifying water can only work if the population uses water that can be treated, for example, tap or bottled water and not collected from natural sources. Research to achieve fortification of different drinking water sources with Ca is fully warranted. Strategies to add Ca to water networks, water wells, bottled water and possibility home devices to increase Ca concentration of water deserve to be investigated and tested to reach the entire population.

## Supplementary material

For supplementary material accompanying this paper visit https://doi.org/10.1017/S1368980020002232. click here to view supplementary material
