# Supplementary material for: Impact of water fortification with calcium on calcium intake in different countries: a simulation study
Source: Public Health Nutr. 2020 Aug 3;25(2):344–57. doi: 10.1017/S1368980020002232 (PMC8883601; doi:10.1017/S1368980020002232)
Supplement: Supplementary file 1 [file S1368980020002232sup001.zip › S1368980020002232supp008.docx]

**Figure S1: Uganda . Distribution of calcium intake before and after fortification. Black line**: Baseline Calcium Mean Intake, **Dashed line**: Estimated Average Requirement (EAR) , **Dotted line:** Upper Limit (UL) of the required intake, **Left Density Distribution**: Distribution of the baseline calcium intake, **Right Density Distribution**: Distribution of the simulated calcium intake after water fortification with 500 mg/L.

**Figure S2: Lao. Distribution of calcium intake before and after fortification. Black line**: Baseline Calcium Mean Intake, **Dashed line**: Estimated Average Requirement (EAR) , **Dotted line:** Upper Limit (UL) of the required intake, **Left Density Distribution**: Distribution of the baseline calcium intake, **Right Density Distribution**: Distribution of the simulated calcium intake after water fortification with 500 mg/L.

**Figure S3: Bangladesh. Distribution of calcium intake before and after fortification. Black line**: Baseline Calcium Mean Intake, **Dashed line**: Estimated Average Requirement (EAR) , **Dotted line:** Upper Limit (UL) of the required intake, **Left Density Distribution**: Distribution of the baseline calcium intake, **Right Density Distribution**: Distribution of the simulated calcium intake after water fortification with 500 mg/L.

**Figure S4: Zambia. Distribution of calcium intake before and after fortification. Black line**: Baseline Calcium Mean Intake, **Dashed line**: Estimated Average Requirement (EAR) , **Dotted line:** Upper Limit (UL) of the required intake, **Left Density Distribution**: Distribution of the baseline calcium intake, **Right Density Distribution**: Distribution of the simulated calcium intake after water fortification with 500 mg/L.

**Figure S5: Argentina. Distribution of calcium intake before and after fortification. Black line**: Baseline Calcium Mean Intake, **Dashed line**: Estimated Average Requirement (EAR) , **Dotted line:** Upper Limit (UL) of the required intake, **Left Density Distribution**: Distribution of the baseline calcium intake, **Right Density Distribution**: Distribution of the simulated calcium intake after water fortification with 500 mg/L.

**Figure S6: Italy. Distribution of calcium intake before and after fortification. Black line**: Baseline Calcium Mean Intake, **Dashed line**: Estimated Average Requirement (EAR) , **Dotted line:** Upper Limit (UL) of the required intake, **Left Density Distribution**: Distribution of the baseline calcium intake, **Right Density Distribution**: Distribution of the simulated calcium intake after water fortification with 500 mg/L.

**Figure S7: USA. Distribution of calcium intake before and after fortification. Black line**: Baseline Calcium Mean Intake, **Dashed line**: Estimated Average Requirement (EAR) , **Dotted line:** Upper Limit (UL) of the required intake, **Left Density Distribution**: Distribution of the baseline calcium intake, **Right Density Distribution**: Distribution of the simulated calcium intake after water fortification with 500 mg/L.
