# Supplementary figures and images for: Impact of water fortification with calcium on calcium intake in different countries: a simulation study
Source: Public Health Nutr. 2020 Aug 3;25(2):344–57. doi: 10.1017/S1368980020002232 (PMC8883601; doi:10.1017/S1368980020002232)

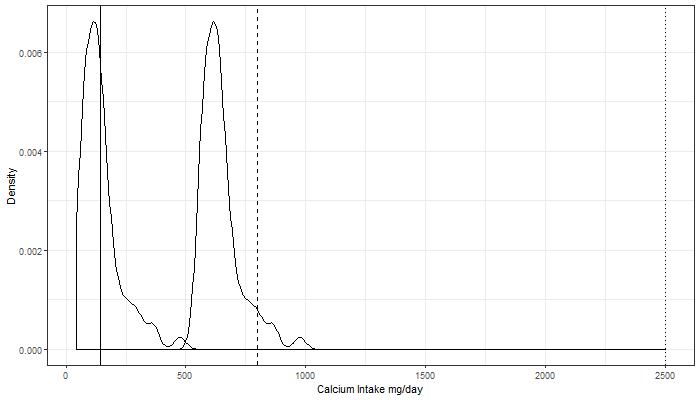

Supplement: Supplementary file 1 [file S1368980020002232sup001.zip › S1368980020002232supp001.jpg]

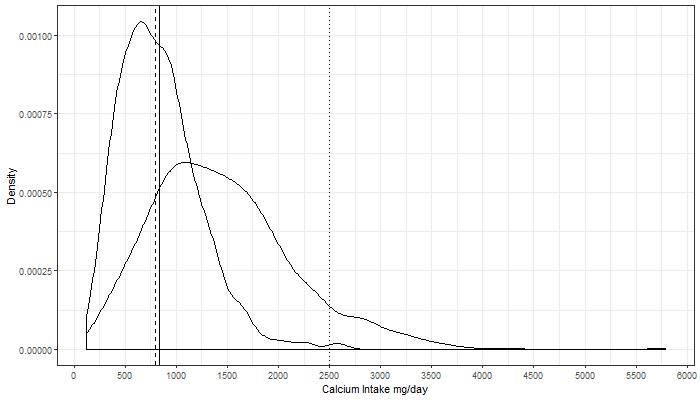

Supplement: Supplementary file 1 [file S1368980020002232sup001.zip › S1368980020002232supp002.jpg]

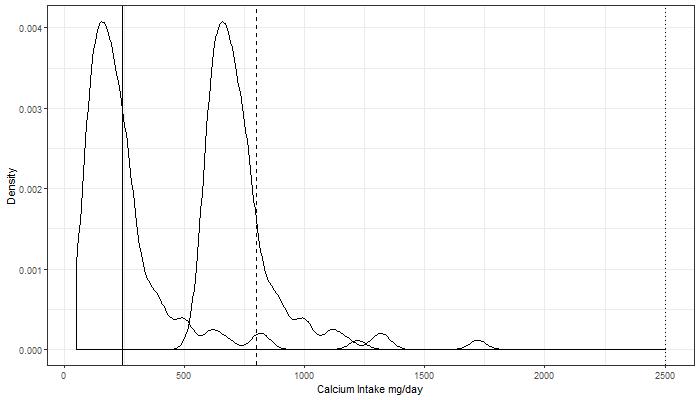

Supplement: Supplementary file 1 [file S1368980020002232sup001.zip › S1368980020002232supp003.jpg]

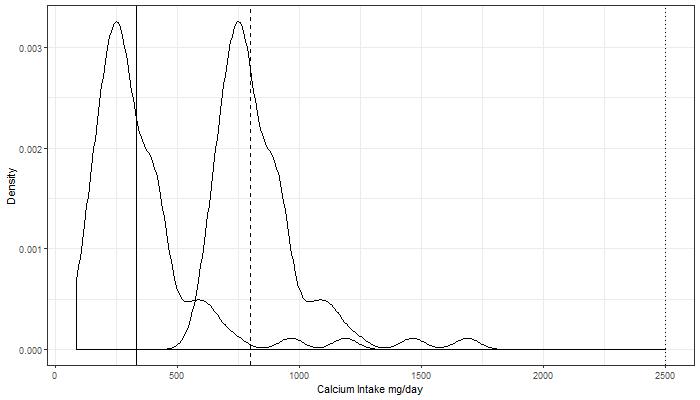

Supplement: Supplementary file 1 [file S1368980020002232sup001.zip › S1368980020002232supp004.jpg]

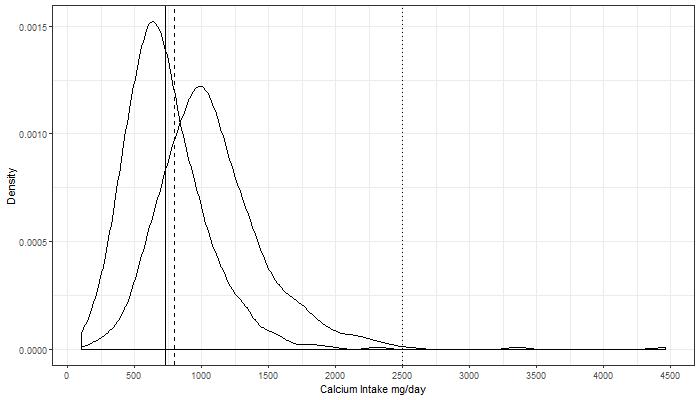

Supplement: Supplementary file 1 [file S1368980020002232sup001.zip › S1368980020002232supp005.jpg]

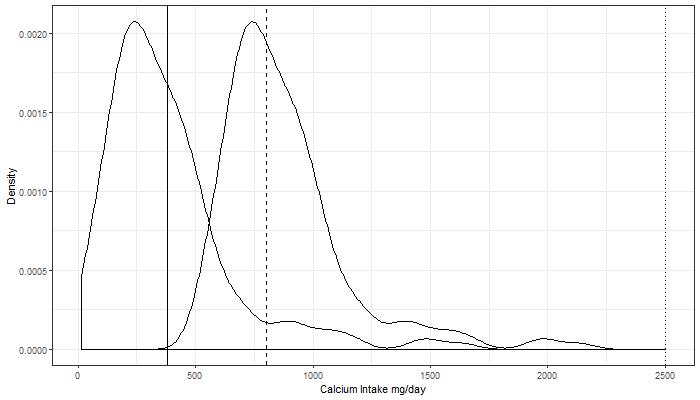

Supplement: Supplementary file 1 [file S1368980020002232sup001.zip › S1368980020002232supp006.jpg]

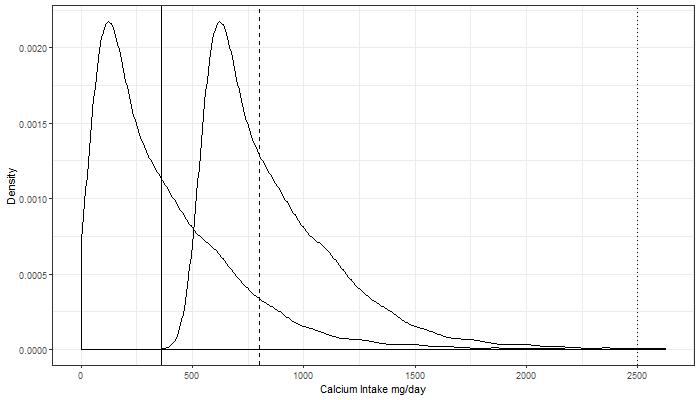

Supplement: Supplementary file 1 [file S1368980020002232sup001.zip › S1368980020002232supp007.jpg]
